# Fully Integrated Ultra-thin Intraoperative Micro-imager for Cancer Detection Using Upconverting Nanoparticles

**DOI:** 10.1007/s11307-022-01710-8

**Published:** 2022-03-21

**Authors:** Hossein Najafiaghdam, Cassio C. S. Pedroso, Nicole A. Torquato, Bruce E. Cohen, Mekhail Anwar

**Affiliations:** 1 Department of Electrical Engineering and Computer Sciences, University of California, Berkeley, CA 94720 USA; 2 Molecular Foundry, Lawrence Berkeley National Laboratory, Berkeley, CA 94720 USA; 3 Division of Molecular Biophysics & Integrated Bioimaging, Lawrence Berkeley National Laboratory, Berkeley, CA 94720 USA; 4 Department of Radiation Oncology, University of California, San Francisco, CA 94158 USA

**Keywords:** Silicon imager, Intraoperative microscopy, Upconverting nanoparticle, Time-resolved imaging

## Abstract

**Purpose:**

Intraoperative detection and removal of microscopic residual disease (MRD) remain critical to the outcome of cancer surgeries. Today’s minimally invasive surgical procedures require miniaturization and surgical integration of highly sensitive imagers to seamlessly integrate into the modern clinical workflow. However, current intraoperative imagers remain cumbersome and still heavily dependent on large lenses and rigid filters, precluding further miniaturization and integration into surgical tools.

**Procedures:**

We have successfully engineered a chip-scale intraoperative micro-imager array—without optical filters or lenses—integrated with lanthanide-based alloyed upconverting nanoparticles (aUCNPs) to achieve tissue imaging using a single micro-chip. This imaging platform is able to leverage the unique optical properties of aUCNPs (long luminescent lifetime, high-efficiency upconversion, no photobleaching) by utilizing a time-resolved imaging method to acquire images using a 36-by-80-pixel, 2.3 mm $\times$ 4.8 mm silicon-based electronic imager micro-chip, that is, less than 100-µm thin. Each pixel incorporates a novel architecture enabling automated background measurement and cancellation. We have validated the performance, spatial resolution, and the background cancellation scheme of the imaging platform, using resolution test targets and mouse prostate tumor sample intratumorally injected with aUCNPs. To demonstrate the ability to image MRD, or tumor margins, we evaluated the imaging platform in visualizing a single-cell thin section of the injected prostate tumor sample.

**Results:**

Tested on USAF resolution targets, the imager is able to achieve a resolution of 71 µm. We have also demonstrated successful background cancellation, achieving a signal-to-background ratio of 8 when performing *ex vivo* imaging on aUCNP-injected prostate tumor sample, improved from originally 0.4. The performance of the imaging platform on single-cell layer sections was also evaluated and the sensor achieved a signal-to-background ratio of 4.3 in resolving cell clusters with sizes as low as 200 cells.

**Conclusion:**

The imaging system proposed here is a scalable chip-scale ultra-thin alternative for bulky conventional intraoperative imagers. Its novel pixel architecture and background correction scheme enable visualization of microscopic-scale residual disease while remaining completely free of lenses and filters, achieving an ultra-miniaturized form factor—critical for intraoperative settings.

**Supplementary Information:**

The online version contains supplementary material available at 10.1007/s11307-022-01710-8.

## Introduction

Real-time intraoperative visualization of microscopic residual disease (MRD) in the tumor bed remains a challenge for image-guided surgeries. This is even more pronounced in today’s surgical practices that have moved towards minimally invasive surgeries. Small, but complex-shaped tumor cavities create a significant challenge for imaging platforms to reach and image hard-to-access resections margins. MRD significantly increases (often doubling) the chance of cancer recurrence across many cancer types [1–3], necessitating substantial additional treatment [4, 5]. In addition to cost, this increases risk of toxicity which can substantially reduce the quality of life [6, 7]. New technologies, such as targeted molecular imaging agents [8–10], miniaturized imaging platforms [11–14], and enhanced optical equipment, have been significant contributors to reducing the incidence of MRD. Nonetheless, MRD still remains a common occurrence [15, 16].

Recent advances in microscopy and intraoperative imaging have resulted in a variety of imaging platforms, creating a viable path for guided resection of tumors [17, 18]. The discovery of novel and more optically efficient fluorophores has partially relieved the stringent optical performance requirements of lenses and optical filters needed for fluorescence microscopy, and as a result, fluorescence microscopes are now able to be scaled down to centimeter-scale dimensions [19–22]. Advancements in waveguide engineering have also reduced the rigidity of these imagers and transformed them to be more flexible and practical for hard-to-access tumor cavities by replacing rigid optical elements with fiber optics to carry optical signals to and from the imaging site [23]. Nonetheless, they still are largely limited by poor light collection efficiency and bending radius—which is a measure of how flexible and adjustable the optical fiber can be. Despite these significant improvements, and their impact on the outcome of cancer surgeries, current intraoperative imaging systems are still reliant on and limited by bulky optical equipment such as filters and lenses that cannot be miniaturized any further. Optical filters used in a fluorescence microscopy imaging system are required to reject the illumination (excitation) light preventing it from saturating the sensor. This poses a significant challenge for commonly used organic fluorophores as the illumination light is many orders of magnitude (often more than 5) stronger than the emitted light, with a wavelength that differs by only a few dozen nanometers from the excitation wavelength [24]. These filters are often thin-film interference filters, which are highly angle dependent [25, 26], necessitating the use of focusing optics, which are challenging to miniaturize.

Conventional fluorescence microscopy also suffers from other disadvantages such as photobleaching [27] of fluorophores and tissue background autofluorescence [28] that cannot be easily mitigated. While tissue autofluorescence cannot be completely eliminated with optical rejection filters, it is however highly attenuated with longer excitation wavelengths and virtually eliminated with excitation in the NIR range and beyond [29]. Nonetheless, organic molecular probes are significantly more efficient with shorter excitation wavelengths. While some organic fluorophores do have the ability to upconvert an NIR excitation into a visible emission—by absorbing two excitation photons and combining the emission energy into a single photon—the power efficiency of these molecular probes remains poor [29].

To address these challenges, a chip-scale imaging platform using in-pixel electronics combined with alloyed upconverting nanoparticles (aUCNP) can be developed to provide a miniaturized and ultra-thin alternative to a conventional, bulky fluorescence imaging system [30, 31]. Chip-scale micro-imagers provide a planar and fully integrated on-chip system that is only a few dozen microns thick and can be easily scaled to any planar form factor, maintaining sensitivity, resolution, and imaging speed due to the parallel imager architecture—a very attractive alternative to large fluorescence instruments. Using integrated micro-fabricated optical structures, these imagers can obviate the need for lenses and provide a standalone imager and broad device integration [30, 32, 33].

To obviate the need for high-performance optical filters, we are leveraging molecular labels that enable time-resolved or lifetime imaging, achieving a significantly smaller form factor and much higher level of integration [34, 35]. The fast decay of the emission of organic fluorophores—only a few nanoseconds—is one of the main reasons why this imaging scheme has remained largely limited [24], particularly for CMOS-based imaging arrays. In addition, tissue autofluorescence lifetimes are often indistinguishable from the desired fluorophore lifetime [36], significantly limiting the achievable signal-to-background ratio (SBR) for time-resolved imaging approaches. Unlike excitation interference, tissue and cellular autofluorescence—which is more pronounced with shorter wavelengths such as UV—cannot be completely blocked or removed by filtering. Longer emitting biomarkers with lifetimes longer than a few microseconds and that are excited at longer wavelengths, in the NIR-I (700–1000 nm) or NIR-II (1000–2000 nm) range, address both of these issues [37].

**Fig. 1.**
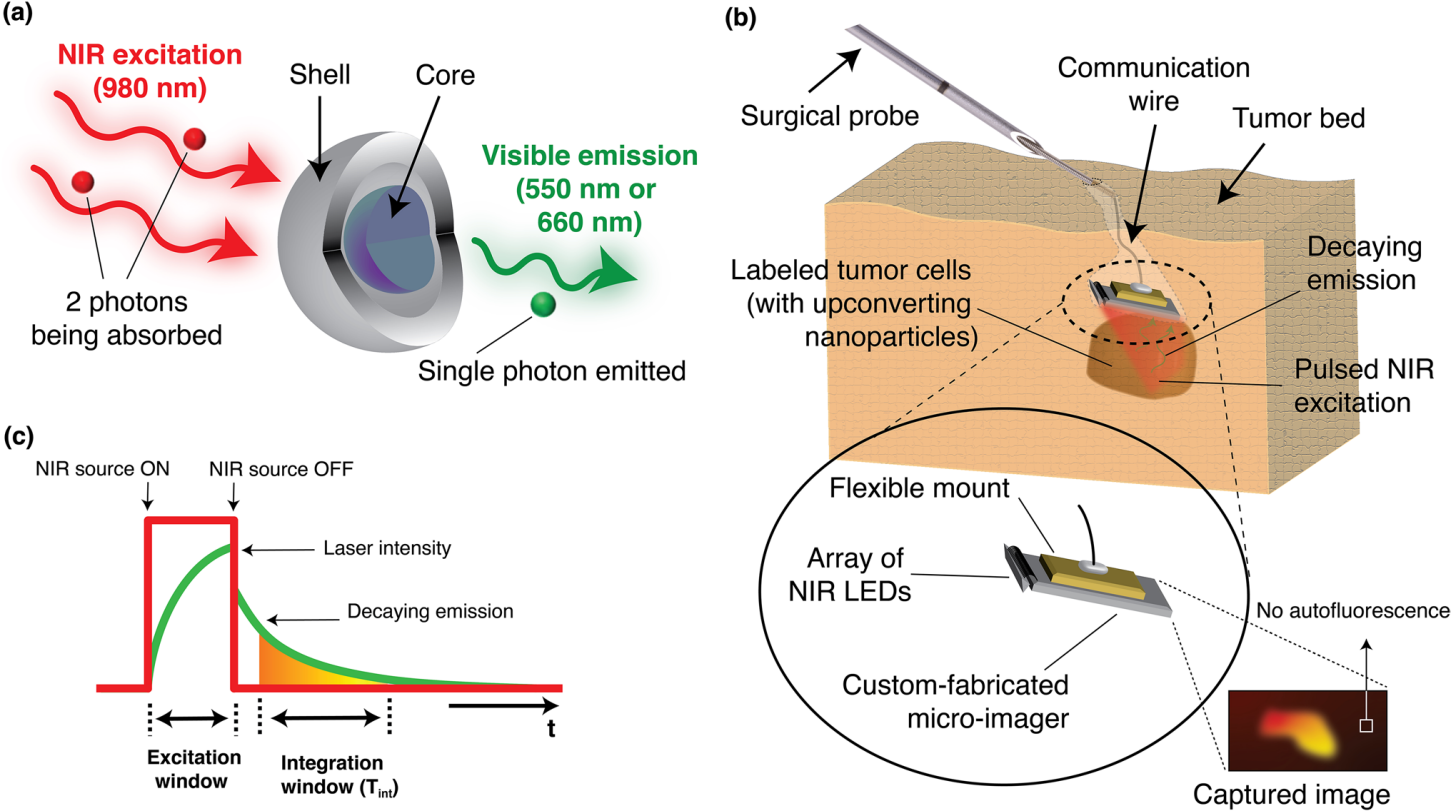
Concept overview of the proposed imaging platform: **a** Illumination scheme of lanthanide-based upconverting nanoparticles. **b** Proposed time-resolved optics-free intraoperative imaging platform for optically guided surgeries. **c** Diagram of time-resolved image acquisition sequence.

Shown in Fig. 1a, lanthanide-based alloyed upconverting nanoparticles are long-lifetime optical probes that combine the energy of two NIR excitation photons into a single higher energy emission photon, effectively upconverting the excitation light into shorter wavelengths (visible) [38, 39]. The efficiency of these upconverting nanoparticles [38, 40] is 10 orders of magnitude higher than that of the best 2-photon fluorophores. Additionally, unlike organic fluorophores, they do not suffer from photobleaching [41] and due to their NIR-I and NIR-II excitation wavelengths and upconversion do not suffer from poor contrast from tissue and cellular autofluorescence. Other nanoparticles such as surface-enhanced Raman scattering (SERS) nanoparticles have also been demonstrated to have the ability to be excited in NIR-I and NIR-II wavelength range and not suffer from photobleaching, but they require several MW/cm^2^ of excitation power to generate detectable optical signal, whereas aUCNPs achieve detection level with only 10 W/cm^2^, which is safe to use in clinical settings [42–44].

Longer wavelengths have the added advantage of a reduced interaction with silicon-based electronics and sensors [45], allowing filter-less use of these upconverting nanoparticles with Complementary Metal Oxide Semiconductors (CMOS) micro-imagers. While the safety exposure limits for humans [46] (~ 200 W/cm^2^ for a 5-ms pulse of 980-nm or 1550-nm light) is insufficient for conventional 2-photon fluorophores, the optical efficiency of the aUCNPs allows for their safe use. Planar and ultra-thin CMOS devices can be used to create intraoperative probes, placed directly in contact with the specimen, and acquiring images with no autofluorescence, as shown in Fig. 1b. However, while silicon-based sensors are less sensitive to NIR wavelengths than visible, they still interact with NIR photons which create optically induced charges throughout the sensor that results in an increased background level in the image. However, unlike tissue autofluorescence, this background can be measured and corrected with auxiliary circuitry integrated within the sensor itself.

In this study, using a fully integrated micro-imager array and engineered nanoparticles, building on our prior aUCNP imaging platform [47], we have developed a next-generation ultra-thin imaging platform that includes a new embedded background adjustment scheme and a novel pixel architecture custom-developed for NIR-range illumination, enabling both higher sensitivity and higher resolution imaging. Using an array of millimeter-sized NIR light sources, similar to [48], and sealed protective packaging, the imaging sensor can be integrated onto surgical instruments or probes—shown in Fig. 1b—to create a standalone micro-imager that would be miniaturized enough to access even small and complex surgical cavities that are otherwise not within reach with larger and bulkier imaging instruments. Our chip-scale imager for intraoperative use leverages the unique optical characteristics of upconverting nanoparticles to perform image acquisition. We eliminated conventional optics by using a time-resolved imaging method, shown in Fig. 1c, made possible by the relatively long emission lifetimes of the aUCNPs. Additionally, we replaced focusing lenses with integrated angle-selective gratings and positioning close to the sample to allow us to achieve a high level of miniaturization. While other works such as [47, 49, 50] have achieved complete or partial elimination of optical filters, they have largely remained angle-sensitive and reliant on a carefully calibrated orientation of excitation beam or required extremely fast lasers (picosecond or femtosecond lasers)—conditions that cannot be guaranteed in intraoperative settings, particularly in small and complex tumor cavities commonly associated with today’s minimally invasive surgical procedures. Despite their stellar performance at UV and visible range, the vast majority of reported time-resolved filter-less imagers are incompatible with universal NIR-I and NIR-II range excitation, as their pixel quenching techniques (used to eliminate excitation background) would degrade significantly with deeper penetration depths of excitation light. The longer wavelength (NIR) light—able to penetrate further deep into the bulk of the silicon sensor—will generate an abundance of long-lifetime charges that can move freely throughout the sensor bulk, and this undesired (and lasting) interference will compromise the sensitivity of the photodiode wells used in quenching techniques. To solve this, we introduce an in-pixel calibration sensor that is relatively insensitive to light emitted from the sample, and instead measures the sensor background. We are therefore able to place the 980-nm excitation source directly above the system and illuminate the area of interest where aUCNPs are to be observed, with no additional angle or orientation considerations, achieving universal NIR imaging compatibility. Thanks to our dual photosensor pixel architecture, our method of background adjustment relies solely on electrical characteristics of silicon-based photosensors and is therefore unaffected by varying angles of incidence. As a result, our imager is now compatible with shorter NIR-I wavelengths such as 980 nm (which can still have significant interaction with silicon), where aUCNPs exhibit a much higher power efficiency than with NIR-II wavelengths, e.g., 1550 nm used in [47]—where silicon is virtually transparent. Increased optical efficiency allows use of a broader laser beam spot, distributing total optical power over a wider area to acquire images of larger areas of interest in a single take, enabling fast and real-time acquisition speeds, and an overall reduced optical power per unit area, ensuring compliance with exposure limits.

The imager was fabricated in a 0.18-µm CMOS technology and measures 2.3 mm by 4.8 mm (and 100-µm thick) and can be thinned down to a thickness of less than 50 µm without performance degradation. In this work, we have characterized the emission profiles of the aUCNPs and its similarities to the NIR-induced background on the sensor that needs to be measured and removed. We then verified the spatial resolution and minimum detectable target clearance using a standard USAF resolution target plate. Finally, we validated the clinical performance and achievable resolution of the imager with *ex vivo* imaging of a mouse prostate tumor intratumorally injected with aUCNPs. To emulate imaging of MRD, the tumor specimen was later sectioned and a 14-µm-thin tumor slice was imaged on the sensor to demonstrate both sensitivity and resolution of the imaging platform on a single-cell thin tissue slice.

## Materials and Methods

### Nanoparticle Synthesis

#### Synthesis of Core/Shell aUCNPs

β-phase NaEr_0.8_Yb_0.2_F_4_ aUCNPs were synthesized with minor modifications of the previously described methodology [38, 51]. The ErCl_3_·xH_2_O (0.32 mmol, 122 mg), YbCl_3_·6H_2_O (0.08 mmol, 31 mg), oleic acid (OA, 3.25 g), and 1-octadececene (ODE, 4 mL) precursors were added into a 50-mL three-neck flask with subsequent heating to 110 °C under vacuum. The solution was stirred for 45 min, yielding clear and homogeneous lanthanide oleates. The flask was cooled to room temperature and filled with N_2_, and sodium oleate (1.25 mmol, 382 mg), NH_4_F (2.0 mmol, 74 mg), and ODE (3 mL) were added. The mixture was placed under vacuum and stirred for 20 min, and then flushed three times with N_2_. The nanocrystal formation occurred after heating to 305 °C under N_2_ for 45 min. A strong stream of air was used to cool rapidly the reaction flask to room temperature. The UCNP dispersion was transferred to a 50-mL centrifuge tube, and 15 mL of EtOH was added to precipitate the nanocrystals. The tube was centrifuged at 3000 g for 5 min to form a white pellet containing the nanocrystals. The supernatant was discarded and the pellet was sonicated in n-hexane (5 mL) to disperse the nanocrystals. The dispersion was centrifuged again at 3000 g for 5 min and transferred to a new 50-mL tube. The purification cycle was repeated, and the UCNPs were stored in 10 mL of hexane with 0.2 % (*v*/*v*) OA under ambient conditions.

Epitaxial NaY_0.8_Gd_0.2_F_4_ shells were grown on aUCNP cores using a layer-by-layer method [38] in the nanocrystal synthesis robot at the Molecular Foundry (WANDA) [52]. The lanthanide oleates was prepared by heating YCl_3_ (0.8 mmol, 156 mg) and GdCl_3_ (0.2 mmol, 53 mg) in oleic acid (4 mL) and 1-octadecene (6 mL) to 110 °C for 30 min under vacuum. The flask was filled with N_2_ and heated to 160 °C for about 30 min, until the solution became clear. Then, the solution was cooled to 110 °C and purged under vacuum for 30 min, to give a 0.10 M solution of 80:20 Y/Gd oleate. In a separate flask, sodium trifluoroacetate NaTFA (2 mmol, 272 mg), 5 mL of OA, and 5 mL of ODE were stirred under vacuum at room temperature for 2 h to complete dissolution of the salt. No solid remained in the flask, giving a 0.20 M NaTFA solution. Core UCNPs in hexane (15 nmol) were stirred under a N_2_ stream to evaporate the solvent. The nanocrystals were redispersed in 10 mL of 2:3 (*v*/*v*) OA/ODE, and the reaction was carried out by a robotic WANDA protocol under N_2_. The core UCNP dispersion was stirred and heated to 280 °C. Then, 0.10 M Ln-OA and 0.20 M NaTFA-OA solutions were injected in alternating cycles into the reaction at 17 μL/s. Each cycle corresponds to the Ln-OA addition, followed by the Na-TFA-OA addition 20 min later to form a single 0.5-nm unit cell layer. The dispersion was stirred for an additional 30 min at 280 °C after the last injection and cooled rapidly to room temperature. The nanoparticles were purified and stored using the procedure described previously for the core UCNPs.

#### Nanoparticle Characterization

To determine aUCNP size and dispersity, dilute dispersions of nanocrystals in hexane were drop cast onto an ultra-thin carbon film on a lacey carbon support, 400 mesh copper TEM grid (Ted Pella) and dried in a fume hood. Images were collected on a Gemini Ultra-55 analytical field emission scanning electron microscope (Zeiss) in dark-field transmission mode under a 30-kV accelerating voltage or on a JEOL 2100-F in HAADF mode under a 200-kV accelerating voltage. Diameters for 100 random nanoparticles were manually designated in ImageJ [38, 41].

#### Aqueous Passivation of Core/Shell UCNPs

Hydrophobic aUCNPs were transferred to water [53] by dissolving 3 mg (0.8 monomer unit per nm^2^ of UCNP surface area) of poly(maleic anhydride-*alt*-1-octadecene) amphiphilic copolymer (PMAO, Sigma-Aldrich) in 1 mL of acetone and 14 mL of CHCl_3_. The mixture was briefly sonicated to obtain a homogeneous solution. UCNPs in n-hexane (0.1 nmol core/shell aUCNPs) were added with stirring, and the solvents were evaporated under a gentle stream of N_2_ overnight. The UCNP/polymer residue was resuspended in 15 mL of 100 mM sodium borate buffer, pH 8.6, with a 10:90 ratio of L-Arginine amide dihydrochloride (14 mg, 57 μmol) to 2-(2-(2-methoxyethoxy)ethoxy)-ethylamine (84 μL, 513 μmol). The flask was sonicated for 4 h in a water bath, and excess polymer was removed by spin dialysis (Amicon Ultra-15, 100 kDa MWCO) washing with 5 $\times$ 7 mL of 100 mM HEPES, pH 7.4. UCNPs in aqueous solution were further purified to remove excess polymer by dialysis (Spectra-Por Float-A-Lyzer G2, 10 mL, 100 kDa MWCO) and concentrated by spin dialysis (Amicon Ultra-15, 100 kDa MWCO) to a final 1 mL dispersion in 20 mM HEPES buffer, pH 7.4.

### Micro-imager Design

The micro-chip imager is designed and fabricated as an ultra-thin imaging array consisting of 2880 pixels where every pixel includes a main photosensor (1444 µm^2^ sensing area) and auxiliary circuitry to help with data and image readout and operation. The micro-chip (measuring only 2.3 mm by 4.8 mm) can be thinned down to a thickness smaller than 50 µm, while maintaining its performance and functionality, as the functional electronic components are confined to the top 10–15 µm. During image acquisition, the optical signal captured by the photosensor is integrated via a highly linear metal-oxide-metal (MOM) capacitor and stored on register capacitors for subsequent readout, resulting in a frame rate of 105 fps.

#### Micro-collimator Integration

Integrating optical structures on chip using CMOS processes are very efficient ways of reducing form factor and achieving higher levels of miniaturization, examples of which have been reported in [30, 32, 54]. We have fabricated angle-selective gratings by directly patterning metal layers in the fabrication process on the chip to create micro-collimators to block obliquely incident light from reaching the sensor, and thus ensuring that the photosensor captures a much more reduced amount of background light.

#### Localized Background Adjustment

**Fig. 2.**
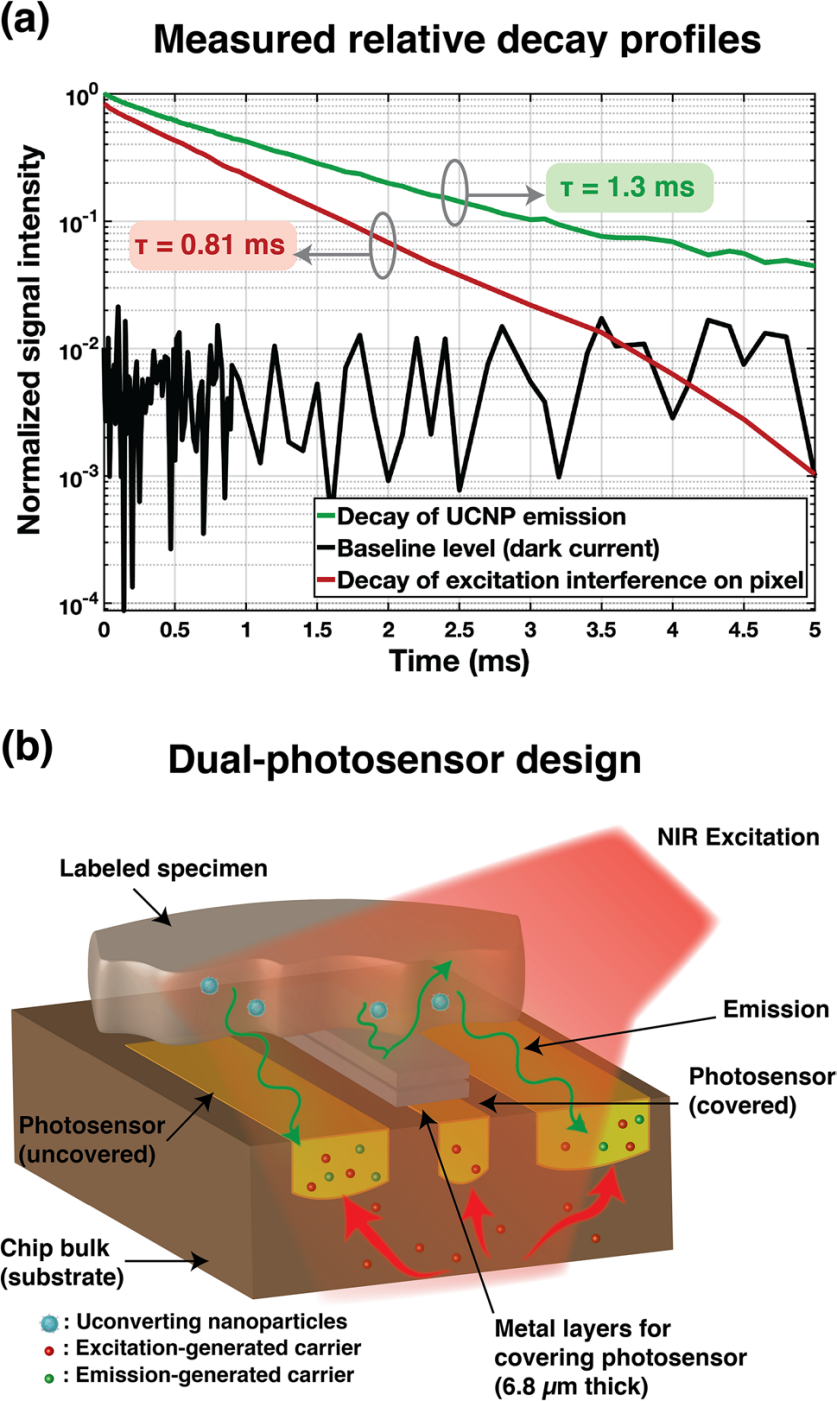
Impact of carriers generated by emission and excitation on pixel (illuminated with 5-ms long pulses of 18 W/cm^2^ 980 nm light): **a** Normalized decay profiles of aUCNP emission, baseline (dark current), and NIR-generated background. **b** Diagram of the dual-photosensor pixel architecture illustrating the main and uncovered photosensor as well as the secondary (covered) photosensor for measuring the local background level.

While NIR-I (and NIR-II) excitation wavelengths do not directly interact with silicon photosensors as much as visible light, they do however have a much higher penetration depth into silicon and therefore still leave a sizeable amount of background on the sensor—relative to the received optical signal—which has to be electronically measured and cancelled out. In order to implement a calibration technique to remove the background of the time-gated excitation light on each pixel, we have first extracted the time domain characteristic of the emission signal captured by the photosensors as well as the amount of background generated by the excitation light, using the setup shown in Suppl. Fig. S1a (see ESM), showing the NIR (980 nm) laser source directly above the micro-chip sensor, before placement of the specimen. A clear quartz optical chamber containing a 0.68-µM dispersion of aUCNPs (in hexane) was then directly placed on the micro-chip (Suppl. Fig. S1b—see ESM), and the chamber was illuminated with 5-ms long pulses of 980-nm light (18 W/cm^2^). The intensity and lifetime of both the emission and background generated by the NIR excitation were measured. The normalized intensities and decays are shown in Fig. 2a, demonstrating that the amount of background is comparable to the emission intensity in both magnitude and lifetime.

Our novel background correction technique involves adding a smaller and fully covered secondary photosensor next to the main one, as shown in Fig. 2b, and locally measure the background level for every pixel and remove it after acquisition. The secondary photosensor is covered with 5 integrated metal layers, creating a 6.8-µm-thick optical shield that will block visible light from reaching the secondary sensor, thus ensuring that the emission signal only reaches the main photosensor. On the other hand, the 980-nm excitation wavelength will penetrate deeper into the uncovered silicon and create unwanted carriers throughout the bulk that will eventually come to the surface to recombine, increasing the background levels on both the main and secondary photosensor—to different extents. Using these local secondary sensors, we can measure the background level on a pixel-by-pixel basis in real time and recover the underlying emission signal by adjusting the baseline level.

Due to limited pixel area, our design minimized the size of the secondary photosensor in favor of keeping the fill factor as high as possible for the main photosensor. The secondary photosensor has been internally positioned to have centroid symmetry and therefore provide a reliable measurement of the local background. While downsizing of the auxiliary photosensor helps with fill factor, the background levels measured on both photosensors no longer exhibit identical profiles, owing to silicon photosensor’s inherent non-linearities. To mitigate this issue, we have performed an initial and one-time extraction and characterization of these profiles by measuring the background for various levels of illumination. Using non-linear curve fitting, we have been able to recover the underlying relationship between the background levels measured on the diode pair and adjust the background on the main photosensor accordingly for each pixel.

### USAF Resolution Target Imaging

To determine the spatial resolution achievable with the proposed ultra-thin micro-imager, we used a standard negative United-States-Air-Force (USAF) resolution target plate to extract the smallest spatial feature this imaging system would be able to properly resolve. The setup used for this experiment is shown in Suppl. Fig. S2a (see ESM), where the USAF resolution target plate is placed directly on the imager, and a quartz optical chamber (with 1 mm optical path) containing 0.68 µM of aUCNPs dispersion is placed atop the plate as a source of emission for the purposes of this experiment. A collimated 980-nm laser, emitting 5-ms-long pulses of 18 W/cm^2^ illuminates the entire system, including the micro-chip and the specimen. The laser is controlled by an external controller module tasked with maintaining proper timing and synchronization between excitation pulses and other relevant control signals. The distance between the emission source (the aUCNP chamber) and the micro-imager is 2.2 mm, 700 µm of which are necessary for protecting the surrounding wirebonds of the imager from mechanical stress, as shown in Suppl. Fig. S2b (see ESM). This separation margin can be virtually eliminated if a more compact electrical contact interface is used for the sensor—e.g., ball-grid array (BGA) contact pads.

To determine the limit of resolution of the imager and extract the smallest detectable target clearance, we imaged 3 line-pairs on the USAF resolution target plate with clearances of 112 µm, 89 µm, and 71 µm, respectively, during which localized background adjustment was performed upon acquiring the image. To determine whether a line-pair has been successfully resolved, we defined “half-width” as the range of pixels in which the amplitude of the emission is more than half the highest intensity, all measured relative to the background level. This metric has been used to determine the spatial resolution of the sensor in this experiment.

### Imaging Intratumorally Injected aUCNPs

All animal experiments are conducted according to protocols approved by the UCSF Animal Care and Use Committee. To demonstrate the performance of our sensor in imaging tumor samples, we injected a mouse prostate tumor with an aqueous solution of polymer-encapsulated upconverting nanoparticles (with a concentration of 250 nM) and subsequently excised that tumor for imaging (see [47] for more details on the sample used). These upconverting nanoparticles were synthesized using a 16 nm NaEr_0.8_Yb_0.2_F_4_ core and a 5-nm NaY_0.8_Gd_0.2_F_4_ shell [38] and the injection volume was 25 µL. The excised mouse tumor was imaged first using a custom-modified IVIS imager equipped with an NIR-I illumination source—980-nm wavelength stabilized single-mode fiber-coupled laser diode—and additional and necessary rejection filters, to verify the colocalization of the specimen and extract the spectrum of the aUCNP emission within the sample. The laser beam size being limited to only a few millimeters in diameter, only areas of interest are illuminated (excited) in this experiment. All emission spectra were extracted under a continuous 45 W/cm^2^ 980 nm illumination.

Upon extracting the emission spectrum, the specimen was then prepared to be imaged on the custom-fabricated micro-imager, as shown in Suppl. Fig. S3 (see ESM). The specimen is directly placed on the imager chip, with no external optical components (i.e., filters and lenses).

The micro-chip is also supported by two connected electronic circuit boards and controlled by an external computer to ensure timing and synchronization is maintained throughout the acquisition process. The controlling computer system is also tasked with communicating with the micro-imager and retrieve and read out acquired images and perform localized background adjustment after acquisition. Additionally, the excitation laser timing is digitally controlled by this control unit to generate pulses of 45 W/cm^2^ NIR light each lasting 5 ms, synchronized with the rest of the acquisition process.

The tumor sample was later sectioned into 14-µm-thin (single-cell layer) sections placed on glass slides, and the resolution and performance of the imaging sensor were evaluated on a sample section of the tumor specimen, using the same experimental setup described previously.

## Results

### USAF Resolution Target

**Fig. 3.**
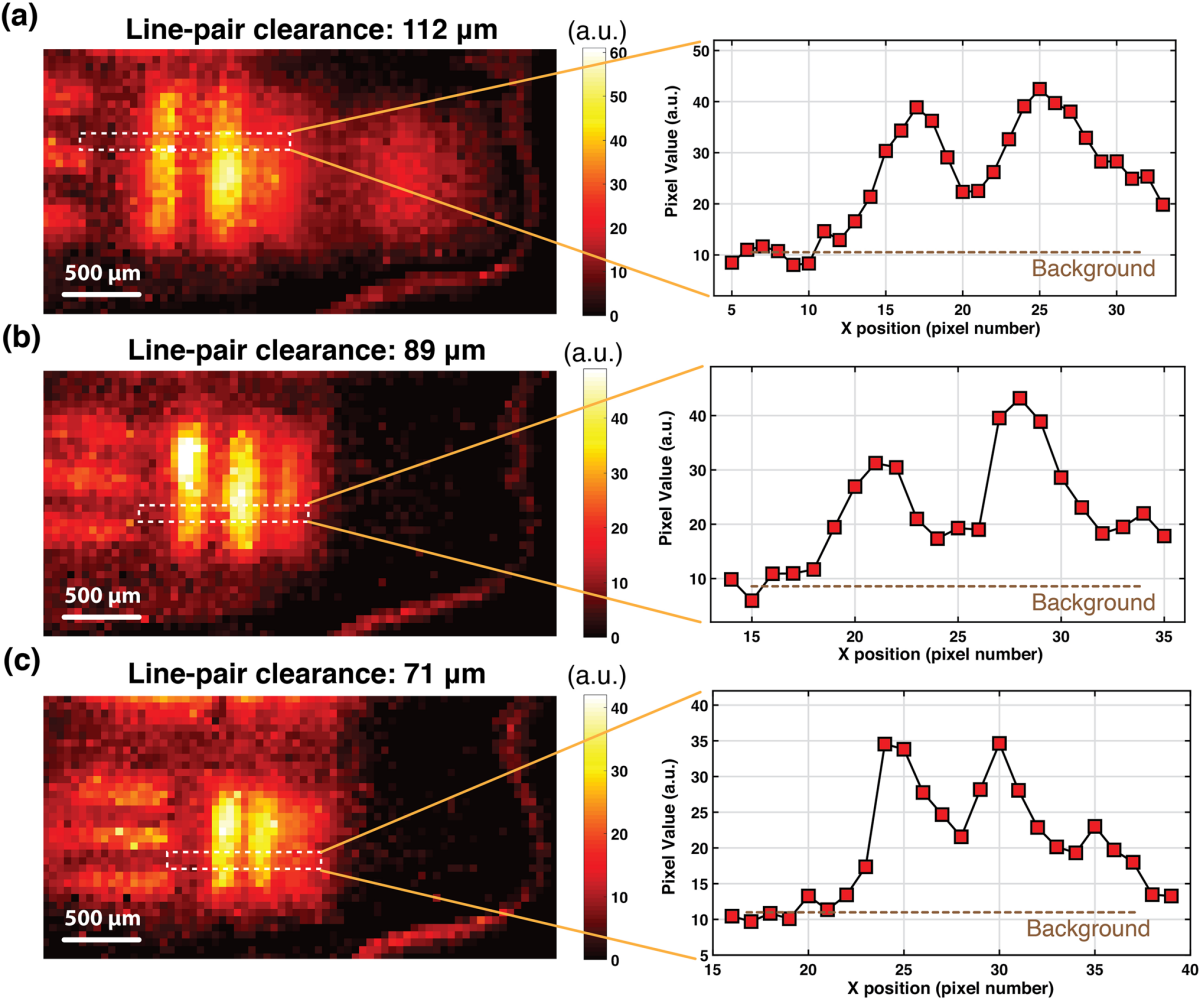
Acquired images and signal intensity cross section of 3 line-pair clearances (distance between line pairs) on the USAF resolution target plate (illuminated with 5-ms-long pulses of 18 W/cm2 980 nm light): **a** Line-pair clearance of 112 µm. **b** Line-pair clearance of 89 µm. **c** Line-pair clearance of 71 µm.

We imaged three line-pairs on the USAF, as described in “USAF Resolution Target Imaging,” and the final resulting images are shown in Fig. 3. Figure 3c illustrates the case for the line-pair with the smallest clearance (71 µm), with a background of 10 a.u., and a value of 35 a.u. for the highest intensity. The half-width range is therefore limited to pixels higher than 22.5 a.u., resulting in structures 71 µm apart be spread by approximately one pixel (55 µm) representing the spatial limit to resolution.

This minimum achievable resolution is largely limited by the accuracy of the background adjustment scheme, and the angle-selective blocking performance of the integrated in-pixel micro-collimators [30].

### Imaging Intratumorally Injected aUCNPs

**Fig. 4.**
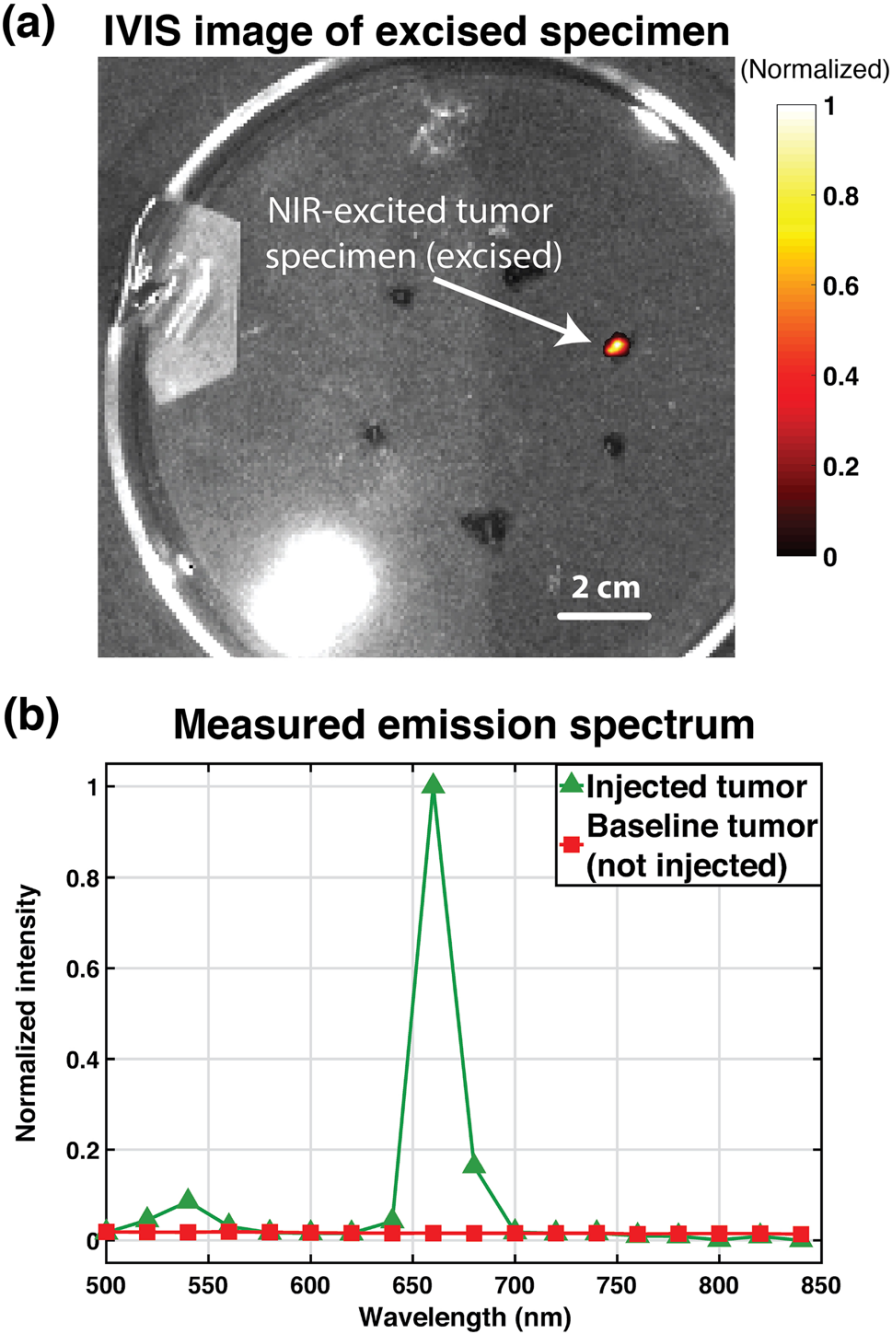
Excised prostate tumor imaging results with IVIS spectrum imager: **a** Image of excised tumor specimen acquired using the 660-nm emission filter (with 20-nm pass band) on the IVIS spectrum imager (excitation provided by custom-modified and external continuous 980-nm laser source). **b** Measured emission of specimen under continuous 980-nm excitation light (22 W/cm^2^).

Figure 4a shows the excised mouse tumor imaged on the IVIS imager and the measured emission of the specimen is plotted in Fig. 4b. The emission spectrum of a mouse tumor without aUCNPs (under 980-nm excitation) is also shown in Fig. 4b as baseline. The emission measured displays the two clear major visible bands of emission of the aUCNPs at 545 nm and 655 nm. The intensity of the measured emission at 545 nm was 8.5 % of the level measured at 655 nm, while the measured SBR in Fig. 4a was 33.5.

**Fig. 5.**
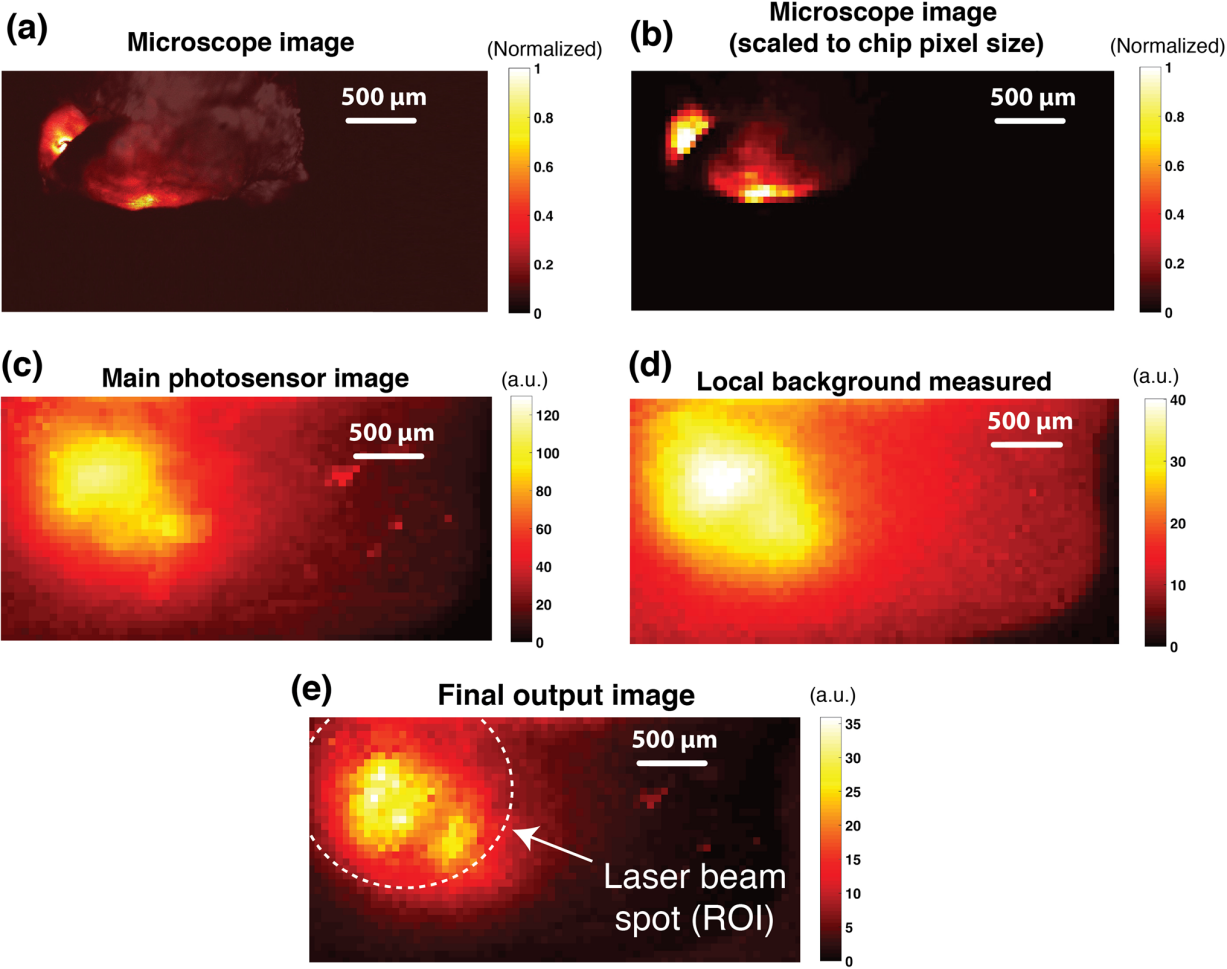
*Ex vivo* experiment images: **a** High-resolution microscope image of excised intratumorally injected prostate tumor (Tint = 1 s). **b** Microscope image of excised prostate tumor with matched pixel pitch (to micro-chip sensor). **c** Main photosensor image of micro-chip sensor. **d** Secondary photosensor image of micro-chip sensor. **e** Image of main photosensor after applying the background adjustment and correction scheme.

The tumor specimen was subsequently imaged with the optics-free custom-fabricated micro-imager using the time-resolved imaging method described in earlier sections. Shown in Fig. 5a is a high-resolution microscope image of the tumor specimen, acquired as a ground-truth. To ensure a fair quantitative comparison of the ground-truth microscope image and the micro-imager result, the microscope image has been resampled to match the pixel size of the custom-fabricated imager, and the resulting image is shown in Fig. 5b.

The image captured using the main photosensors in each pixel is shown in Fig. 5c displaying a significantly high level of background, virtually masking the underlying emission signal from the specimen, highlighting one of the most critical limitations of prior works such as [47, 50]. To correct this background level, an additional acquisition is performed to capture the data from the secondary (fully covered) photosensors and extract the locally measured (pixel-level) background (Fig 5(d)). These measured local background levels are then used to adjust the background on the main photosensor image and recover the underlying emission of the specimen. Figure 5e shows the main photosensor image after background correction, revealing the emission signal, with maximum measured signal of 35 a.u. and an SBR of approximately 8. This new dual-photosensor architecture was able to correct more than 80 a.u. of background measured in Fig. 5c—where the effective SBR was 35/80 or 0.4—enhancing the SBR by 20×, with no additional optics or increase in form factor. Figure 5e demonstrates the correlation of the micro-chip image with the microscope ground-truth data shown in Fig. 5b.

**Fig. 6.**
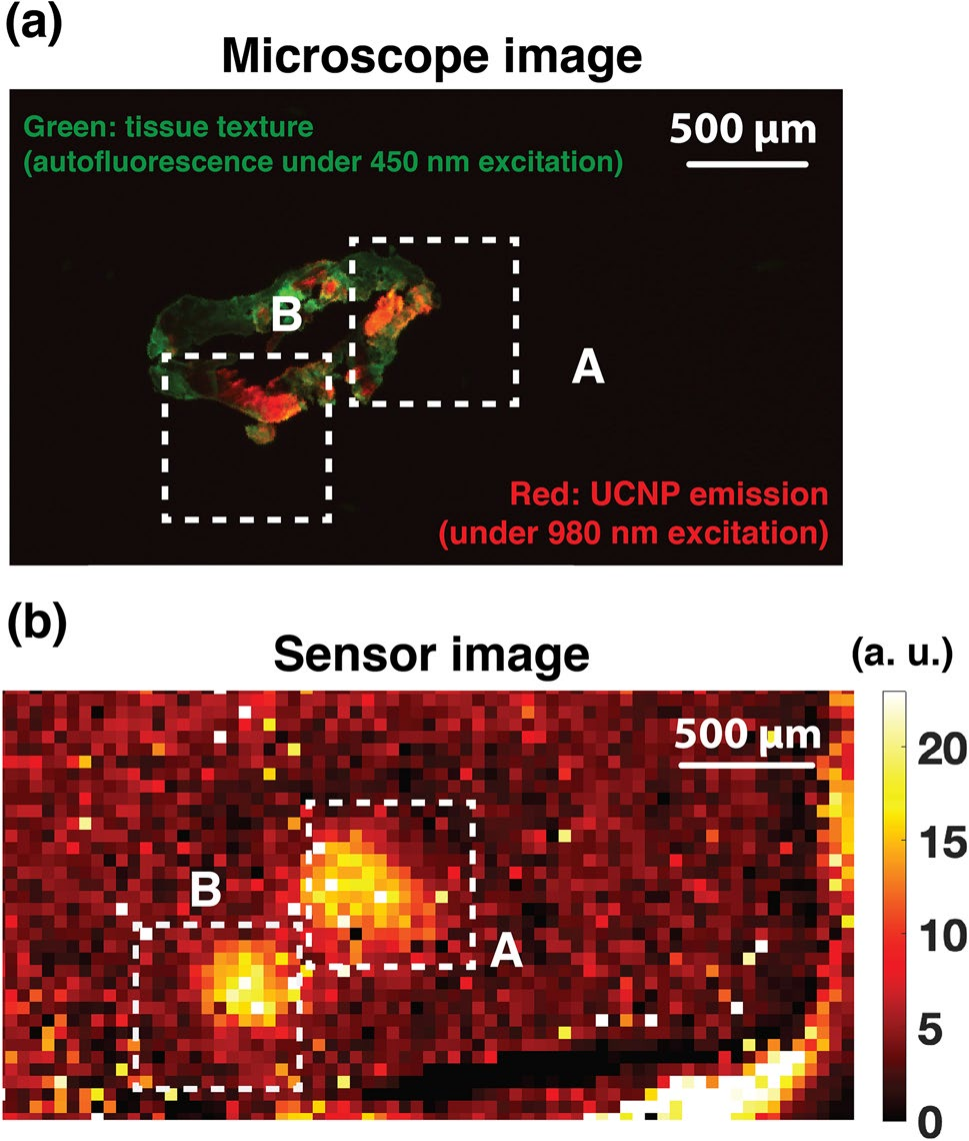
Single-cell thin-layer imaging experiment: **a** Composite microscope image of a 14-µm-thin section of intratumorally injected prostate tumor specimen, with the green color representing tissue texture obtained using autofluorescence under a 450-nm excitation light and red representing the UCNP emission captured using a continuous 45 W/cm^2^ 980 nm excitation (Tint = 1 s). **b** Background-adjusted image of the 14-µm-thin section captured on the micro-chip under pulsed 980-nm excitation (45 W/cm^2^).

To image emulated MRD, 14-µm-thin section of this tumor specimen was extracted and placed on a glass slide and visualized on the microscope. The aUCNP emission was obtained under a continuous 980-nm excitation (45 W/cm^2^) and an image of the tissue itself was captured using tissue autofluorescence under a 450-nm excitation light. A composite overlay of the high-resolution images obtained on the microscope is shown in Fig. 6a, illustrating two distinct sites with a higher concentration of aUCNPs. The slide is then directly placed on the micro-chip, as described in the setup in Suppl. Fig. S3 (see ESM). The image captured by the micro-chip is shown in Fig. 6b and is obtained using a pulsed 45 W/cm^2^ 980 nm excitation light. The two areas of aUCNP are highlighted in Fig. 6a and b, corresponding to the regions identified on the microscope image. Each of the emission site in zones A and B is approximately 150 µm in diameter and contains about 200 cells. The imaging sensor was able to resolve both sites with a signal-to-background ratio of 4.3 (or 21/4.9). The reduced SBR is explained by the reduced signal intensity in the 14-µm section of the specimen compared to the 2-mm-thick unsectioned sample.

## Discussion

In this work, we presented a new platform for intraoperative imaging, dispensing with conventional bulky optical devices in favor of completely optics-free silicon-based contact imager arrays. This is made possible by a synergistic combination of aUCNPs with unique optical properties—high optical efficiency, long decay lifetimes, upconversion capability, and no photobleaching—and custom integrated circuit (IC) design with a time-resolved array and in-pixel background subtraction. Unlike other inorganic molecular marker such as quantum dots, these lanthanide-based UCNPs do not contain elements known to cause toxicity, and previous animal injections have been safely demonstrated in [38, 47, 55], opening the door to clinical translation. In addition to that, they are also able to be conjugated to antibodies enabling molecular targeting to cells, providing cellular level contrast and selectivity [51]. Table 1 includes a comparison of the two main types of time-resolved biomedical sensors: systems requiring optics [35, 56] and their optics-free counterparts [47, 57]. While other works, as seen in Table 1, have also been able to achieve filter- and lens-less acquisition using beam orienting and long (> 1.5 µm) NIR excitation wavelength [47] or correlated samplings of single-photon avalanche diodes [57], their performance will significantly degrade when directly exposed to or excited with NIR-I light such as 980 nm, due to the penetration depth of NIR-I and the generation of long-lasting (millisecond range) background that will mask the signal to be detected. As a result, they are incompatible with universal (NIR-I and NIR-II) imaging. Our novel dual-photosensor design architecture, however, ensures a relaxed limit on the orientation of the excitation source, and additionally, the background cancellation is no longer wavelength-specific and can be easily modified to be compatible with any given NIR wavelengths ($\lambda$< 2 µm), enabling optics-free imaging of targeted microscopic residual disease sites with upconverting nanoparticles in clinical settings.

**Table 1:** Comparison table of time-resolved biomedical sensors

	IEEE TED’12 [35]	Theranostics’19 [47]	JSSC’12 [56]	JSSC’19 [57]	This work
Pixel array	256 $\times$ 256	36 $\times$ 80	32 $\times$ 32	192 $\times$ 128	36 $\times$ 80
Frame rate	15 fps	137 fps	20 fps	18.6 kfps	105 fps
Array size (mm $\times$ mm)	-	4.9 $\times$ 2.5	4 × 4	3.2 $\times$ 2.4	2.3 $\times$ 4.8
Fill factor	4.6 %	64 %	37 %	13 %	47 %
Excitation wavelength	440 nm	1550 nm (NIR-II)	~ 610 nm	685 nm	any $\lambda$ < 2 µm
Optics used	Filter and lens	None	Filter and lens	None	None
Noise (rms full scale)	-	0.4 %	4.6 %	-	0.36 %

The proposed imaging platform demonstrated being able to resolve targets with a clearance of 71 µm, achieving enough spatial resolution for intraoperative imaging of microscopic disease to guide cancer surgery, such as identification of tumor or critical normal tissue structures.

The sensor was also able to resolve regions of aUCNPs on a 2-mm-thick tumor specimen directly placed on the micro-chip with no optics or focusing lenses—directly illuminated with an NIR-I (980 nm) laser—and achieved a signal-to-background ratio of 8. The new pixel architecture with in-pixel background measurement and subtraction was able to reduce the background by more than 20 × , highlighting the critical role of the novel background adjustment scheme. This performance was also maintained when imaging a single 14-µm section of the described specimen, and the background correction scheme resulted in an SBR of 4.3, with the reduction in SBR due to the smaller amounts of aUCNPs per area and therefore a lower signal intensity. This demonstrates the applicability of our sensor to image microscopic deposits of disease, opening the door to real true intraoperative imaging in a compact form factor.

This imager array achieved real-time frame rates (105 fps) and an output (measured) noise level of 0.36 % full scale rms—where full scale is the count limit of the pixel, i.e., 600 a.u. Most of this noise is contributed by the pixel circuitry (i.e., in-pixel amplifier) but can be further attenuated by temporal averaging (averaging consecutive and repeated images), made possible by the fact that aUCNPs do not photobleach. A fill factor of 47 % was also achieved, with losses mainly attributed to in-pixel readout circuitry as well as the additional secondary photosensor.

Further improvements of this platform may include the use of external planar micro-gratings to enhance image sharpness and optimization of the secondary photodiode size and shape to maximize emission signal captured on the main photodiode while still providing a reliable measurement of the local background. The recent demonstrations of molecularly targeted aUCNPs can unlock the potential for molecularly guided intraoperative optical navigation using this platform.

## Conclusion

We have demonstrated a chip-scale optics-free (filter- and lens-less) intraoperative imaging platform that utilizes the optical properties of lanthanide-based upconverting nanoparticles as molecular probes and leverages their uniquely long emission decay times and long excitation wavelength (980 nm) to perform time-resolved imaging in an environment where no tissue autofluorescence or photobleaching is present. The core of this platform is an ultra-thin (less than 100-µm thick) millimeter-scale 36-by-80 pixel-array fabricated in CMOS technology and includes a novel dual-photosensor pixel architecture capable of measuring and correcting the NIR-induced background interference generated by the time-gated excitation light. The resolution of this imaging platform was measured using standard USAF resolution targets and achieved a minimum resolution of 71 µm. We also evaluated the performance of the imaging platform using *ex vivo* imaging of mouse prostate tumor specimen intratumorally injected with aUCNPs, where the dual-photosensor design was able to reduce the background level by approximately 20 times and achieve a signal-to-background ratio of 8. The imager also demonstrated the ability to resolve single-layer cell clusters as small as 200 cells with a signal-to-background ratio of 4.3, opening the door to highly sensitive intraoperative imaging with a minimal form factor chip-scale imager.

## Supplementary Information

Below is the link to the electronic supplementary material.

Supplementary file1 (DOCX 2622 KB)
