# Supplementary material for: Fully Integrated Ultra-thin Intraoperative Micro-imager for Cancer Detection Using Upconverting Nanoparticles
Source: Mol Imaging Biol. 2022 Mar 21;25(1):168–79. doi: 10.1007/s11307-022-01710-8 (PMC9970948; doi:10.1007/s11307-022-01710-8)
Supplement: Supplementary file 1 — Supplementary file1 (DOCX 2622 KB) [file 11307_2022_1710_MOESM1_ESM.docx]

# **Electronic Supplementary Material**

# Fully Integrated Ultra-Thin Intraoperative Micro-Imager for Cancer Detection Using Upconverting Nanoparticles

Journal: Molecular Imaging and Biology

Hossein Najafiaghdam^1[*]^, Cassio C.S. Pedroso^2^, Nicole A. Torquato^3^, Bruce E. Cohen^4^, Mekhail Anwar^5[*]^

[*] Corresponding authors

[1] Department of Electrical Engineering and Computer Sciences, University of California, Berkeley, CA 94720 USA (e-mail: hossein_najafi@berkeley.edu, +1-510-816-4455)

[2] Molecular Foundry, Lawrence Berkeley National Laboratory, Berkeley, CA 94720 USA (email: [CCSPedroso@lbl.gov](mailto:CCSPedroso@lbl.gov))

[3] Molecular Foundry, Lawrence Berkeley National Laboratory, Berkeley, CA 94720 USA (email: ntorquat@ucsd.edu)

[4] Molecular Foundry and the Division of Molecular Biophysics & Integrated Bioimaging, Lawrence Berkeley National Laboratory, Berkeley, CA 94720 USA (email: becohen@lbl.gov)

[5] Department of Radiation Oncology, University of California, San Francisco, CA 94158 USA and Department of Electrical Engineering and Computer Sciences, University of California, Berkeley, CA 94720 USA (mekhail.anwar@ucsf.edu)


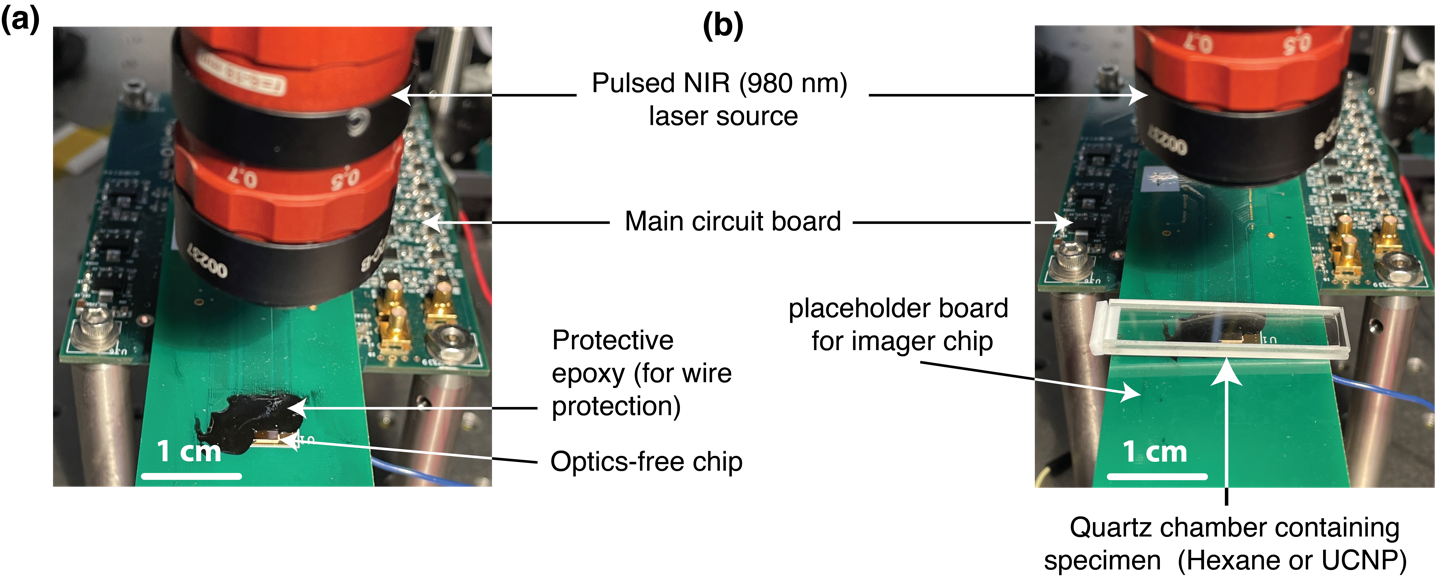


Suppl. Fig. S1. Experimental setup for decay profile extraction of aUCNP emission, baseline and NIR-generated background: (a) imaging platform, including NIR laser source, micro-chip sensor and circuit boards. (b) 1-mm thick clear quartz optical chamber containing 0.68 µM dispersion of aUCNPs placed on sensor.

**
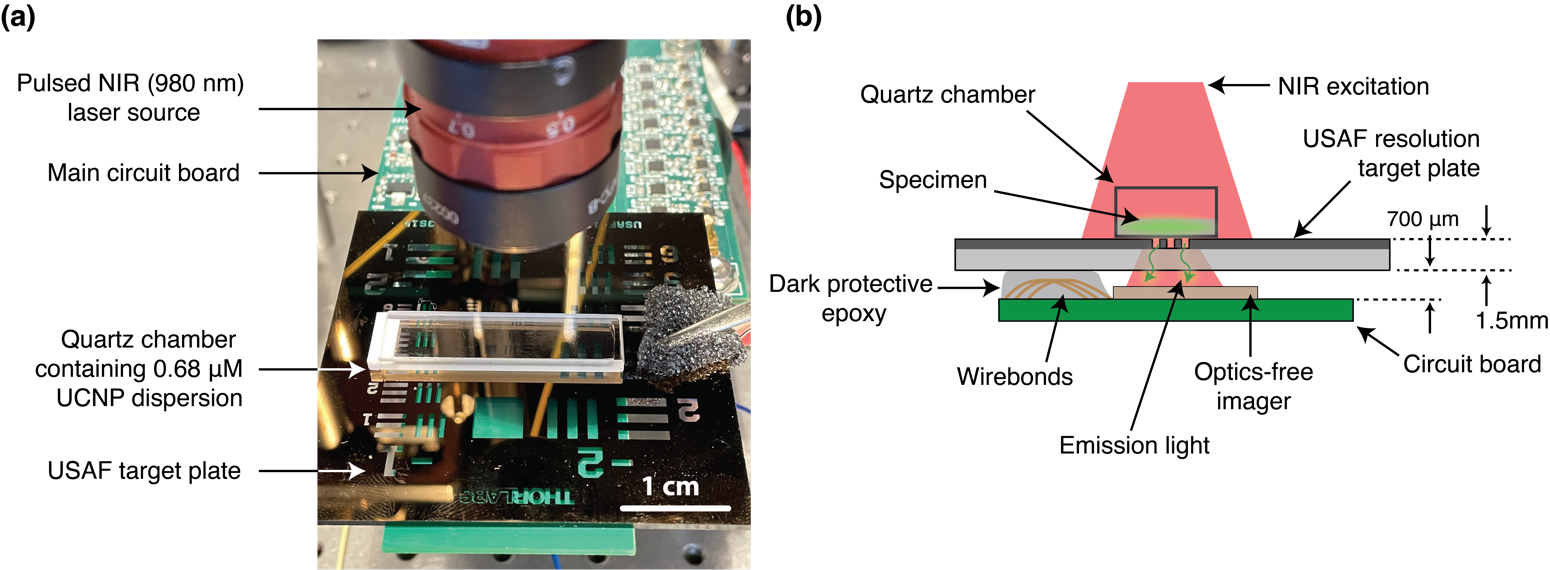
**

Suppl. Fig. S2. Experiment setup for minimum detectable target clearance measurement (using a negative standard USAF resolution target plate): (a) imaging platform with 1-mm thick clear quartz chamber containing 0.68 µM dispersion of aUCNPs placed on the USAF resolution target plate, directly mounted on the sensor. (b) cross-section of the imaging setup.


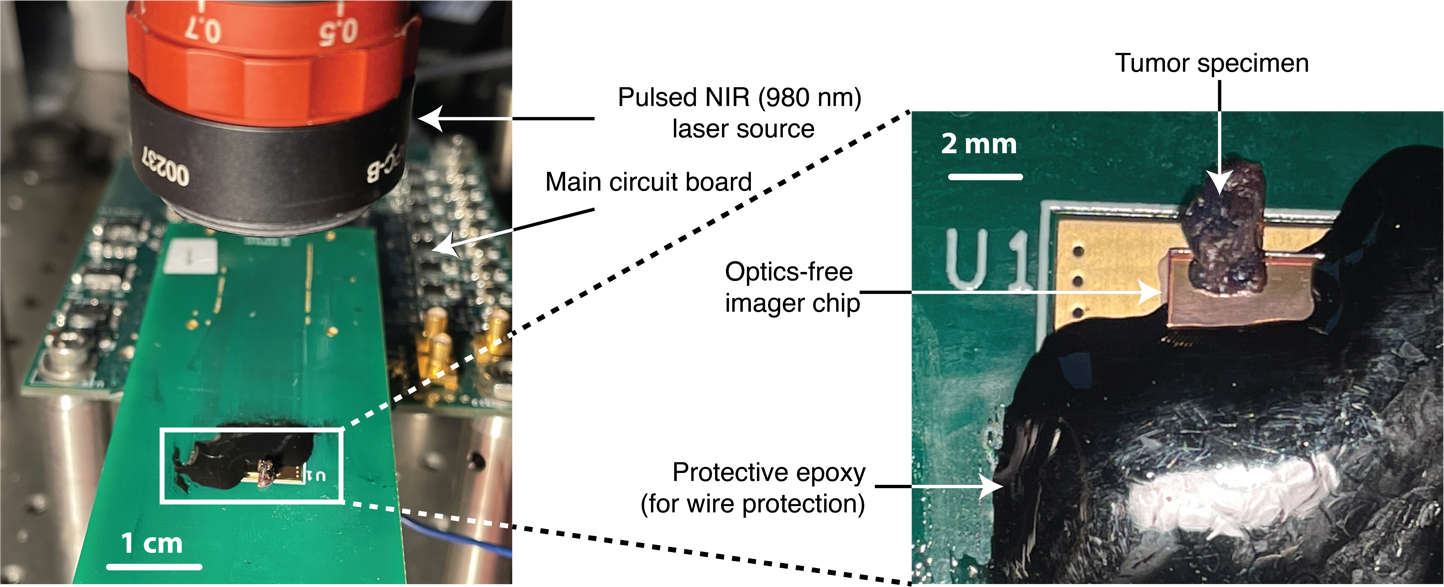


Suppl. Fig. S3. Experiment lab setup for *ex vivo* imaging of intratumorally-injected prostate tumor with alloyed upconverting nanoparticles (illuminated with 5-ms long pulses of 45 W/cm^2^ 980 nm light).
